# Isolated small airways obstruction predicts future chronic airflow obstruction: a multinational longitudinal study

**DOI:** 10.1136/bmjresp-2023-002056

**Published:** 2023-11-20

**Authors:** Ben Knox-Brown, James Potts, Valentina Quintero Santofimio, Cosetta Minelli, Jaymini Patel, Najlaa Mohammed Abass, Dhiraj Agarwal, Rana Ahmed, Padukudru Anand Mahesh, Jayaraj BS, Meriam Denguezli, Frits Franssen, Thorarinn Gislason, Christer Janson, Sanjay K Juvekar, Parvaiz Koul, Andrei Malinovschi, Asaad Ahmed Nafees, Rune Nielsen, Stefanni Nonna M Paraguas, Sonia Buist, Peter GJ Burney, Andre F S Amaral

**Affiliations:** 1 National Heart and Lung Institute, Imperial College London, London, UK; 2 NIHR Imperial Biomedical Research Centre, London, UK; 3 College of Medicine, Alshaab Teaching Hospital, Bahri University, Khartoum, Sudan; 4 Vadu Rural Health Program, KEM Hospital Pune Research Centre, Pune, India; 5 The Epidemiological Laboratory, Khartoum, Sudan; 6 Respiratory Medicine, JSS Medical College, Mysore, Karnataka, India; 7 Faculte de Medecine de Sousse, Universite de Sousse, Sousse, Tunisia; 8 Respiratory medicine, Maastricht University Medical Centre+, Maastricht, Netherlands; 9 Research and Education, CIRO, Horn, Netherlands; 10 Department of Sleep, Landspitali University Hospital, Reykjavik, Iceland; 11 Faculty of Medicine, University of Iceland, Reykjavik, Iceland; 12 Department of Medical Sciences Respiratory, Allergy and Sleep Research, Uppsala University, Uppsala, Sweden; 13 Sher-i-Kashmir Institute of Medical Sciences, Srinagar, Jammu and Kashmir, India; 14 Department of Community Health Sciences, The Aga Khan University, Karachi, Sindh, Pakistan; 15 Department of Thoracic Medicine, University of Bergen, Bergen, Hordaland, Norway; 16 Philippine College of Chest Physicians, Quezon City, Philippines; 17 Philippine Heart Center, Quezon City, Manila, Philippines; 18 Oregon Health & Science University, Portland, Oregon, USA

**Keywords:** COPD epidemiology, Lung Physiology

## Abstract

**Background:**

Chronic airflow obstruction is a key characteristic of chronic obstructive pulmonary disease. We investigated whether isolated small airways obstruction is associated with chronic airflow obstruction later in life.

**Methods:**

We used longitudinal data from 3957 participants of the multinational Burden of Obstructive Lung Disease study. We defined isolated small airways obstruction using the prebronchodilator mean forced expiratory flow rate between 25% and 75% of the forced vital capacity (FVC) (FEF_25–75_) if a result was less than the lower limit of normal (<LLN) in the presence of a normal forced expiratory volume in 1 s to FVC ratio (FEV_1_/FVC). We also used the forced expiratory volume in 3 s to FVC ratio (FEV_3_/FVC) to define small airways obstruction. We defined chronic airflow obstruction as post-bronchodilator FEV_1_/FVC<LLN. We performed mixed effects regression analyses to model the association between baseline isolated small airways obstruction and chronic airflow obstruction at follow-up. We assessed discriminative and predictive ability by calculating the area under the receiver operating curve (AUC) and Brier score. We replicated our analyses in 26 512 participants of the UK Biobank study.

**Results:**

Median follow-up time was 8.3 years. Chronic airflow obstruction was more likely to develop in participants with isolated small airways obstruction at baseline (FEF_25-75_ less than the LLN, OR: 2.95, 95% CI 1.02 to 8.54; FEV_3_/FVC less than the LLN, OR: 1.94, 95% CI 1.05 to 3.62). FEF_25-75_ was better than the FEV_3_/FVC ratio to discriminate future chronic airflow obstruction (AUC: 0.764 vs 0.692). Results were similar among participants of the UK Biobank study.

**Conclusion:**

Measurements of small airways obstruction can be used as early markers of future obstructive lung disease.

**WHAT IS ALREADY KNOWN ON THIS TOPIC**

- Small hospital-based studies and studies of symptomatic smokers have suggested that isolated small airways obstruction predicts future chronic airflow obstruction.

**WHAT THIS STUDY ADDS**

- In general populations, individuals with isolated small airways obstruction are at greater risk of lung function decline and development of chronic airflow obstruction.

**HOW THIS STUDY MIGHT AFFECT RESEARCH, PRACTICE OR POLICY**

- These findings will lead to more research into the role of the small airways in chronic obstructive pulmonary disease and how to target them to prevent disease. They will also raise awareness of clinicians to the potential benefit of keeping record of patients’ forced expiratory flows, in addition to other more common spirometry measures.

## Introduction

Chronic obstructive pulmonary disease (COPD) is a heterogeneous condition associated with reduced lifespan,^1^ increased disability^2^ and a greater dependence on healthcare services.^3^ A primary feature of COPD is chronic airflow obstruction, which is defined by an abnormal postbronchodilator forced expiratory volume in 1 s to forced vital capacity ratio (FEV_1_/FVC).^4^ The FEV_1_/FVC ratio is non-specific, reflecting the presence of airflow obstruction in both the large and small airways. However, in early disease, damage is largely confined to the small airways.^5^

Parameters that are universally measured by spirometry devices but seldom reported are the mean forced expiratory flow rate between 25% and 75% of the forced vital capacity (FEF_25-75_) and the forced expiratory volume in three seconds as a ratio of the forced vital capacity (FEV_3_/FVC). There is increasing interest in the use of these parameters to identify airflow obstruction in the small airways,^6^ and studies in ever smokers have shown that individuals with atypical measurements for these parameters have evidence of functional small airways disease, gas trapping and emphysema on CT, even when lung function is normal according to traditional measurement indices (ie, isolated small airways obstruction).^7–10^ Despite these findings, it is still widely believed that these parameters are neither sensitive nor specific to changes within the small airways.^11^

Studies in selected clinical populations and symptomatic ever smokers have found that individuals with isolated small airways obstruction are at greater risk of developing chronic airflow obstruction in later life.^8 12^ To the best of our knowledge, no study has attempted to determine whether this is also true in general populations. Using longitudinal data from the multinational Burden of Obstructive Lung Disease (BOLD) study, we aimed to investigate if having isolated small airways obstruction at baseline was associated with progression to chronic airflow obstruction at follow-up and to compare results for two different definitions of small airways obstruction. We also aimed to replicate our findings using data from the UK Biobank study.

## Methods

### Main study

#### Study population

The BOLD study is a multinational observational cohort study whose protocol has been published previously.^13 14^ Between January 2003 and December 2016, non-institutionalised adults ≥40 years of age were recruited from 41 municipalities, across 34 countries. Site-specific sampling strategies were implemented to randomly recruit representative samples of the populations studied. Participants from 18 sites were then followed up between January 2019 and October 2021. For the present study, participants were included if they had completed the study core questionnaire and had acceptable spirometry at both baseline and follow-up. Participants were excluded if they had a contraindication for lung function testing at either visit.

#### Procedures

Demographic data and information on respiratory symptoms, health status and exposure to potential risk factors were collected by trained staff, who administered standardised questionnaires translated into the local language. Lung function, including FEV_1_, FVC, FEV_3_ and FEF_25-75_, was measured using the ndd EasyOne Spirometer (ndd Medizintechnik AG, Zurich, Switzerland), before and 15 min after inhaled salbutamol (200 μg). Spirograms were centrally reviewed and assigned a quality score based on acceptability and reproducibility criteria.^15^

#### Definitions of spirometric abnormalities

At baseline, we defined isolated small airways obstruction as a prebronchodilator FEF_25-75_ less than the lower limit of normal (LLN), with an FEV_1_/FVC ratio equal to or greater than the LLN. Due to the perceived lack of clinical utility for FEF_25-75_ and its large between subject variation in normal populations,^11 16^ we investigated a second parameter, the FEV_3_/FVC ratio using the same definition. At follow-up, we defined chronic airflow obstruction as postbronchodilator FEV_1_/FVC ratio below the LLN. To calculate the LLN, we used reference equations for European Americans in the third US National Health and Nutrition Examination Survey.^17 18^

#### Statistical analysis

We calculated the incidence rate of chronic airflow obstruction per 1000 person years. To estimate the association between having isolated small airways obstruction at baseline and chronic airflow obstruction at follow-up, we performed multilevel (mixed effects) logistic regression analyses to account for clustering by study site. We also used multilevel linear regression to estimate the association between isolated small airways obstruction and postbronchodilator FEV_1_/FVC ratio as a continuous measure. We initially considered all potential risk factors for chronic airflow obstruction,^19^ however, as risk factors for progression from isolated small airways obstruction to chronic airflow obstruction are largely unknown, we then used a backward elimination procedure, keeping only those variables that were significant in the final model: sex (male/female), age (years), body mass index (BMI; kg/m²), smoking status (never/former/current), and pack years of smoking. We modelled the association of isolated small airways obstruction with chronic airflow obstruction using a random slope to allow the magnitude of the association to vary by study site. We did not directly model the effect of follow-up time as this was determined at site level. However, to check for effect modification, we performed stratified analyses in those with less than 5-year follow-up and those with equal to or greater than 5-year follow-up. We also performed stratified analyses by sex to investigate possible effect modification. Finally, we performed a sensitivity analysis on never smokers to investigate any residual confounding due to smoking. All analyses were conducted using inverse probability weights^20^ to account for missing data at follow-up.

Receiver-operating characteristic curves were constructed, and the area under the curve (AUC) calculated for both FEF_25-75_ and the FEV_3_/FVC ratio to determine their sensitivity and specificity in predicting chronic airflow obstruction. The AUC values of the two parameters were compared as previously described.^21^ In addition, we evaluated the incremental value of both parameters to determine if they conveyed an improvement in classification accuracy over a model containing age, sex, BMI and smoking history.^22^ To assess the overall predictive performance of the parameters, we calculated the Brier score, which ranges between 0 and 1, with 0 indicating a perfect prediction and 1 a poor predictive ability.^23^ All results were considered significant if the p value was below 0.05. Analyses were performed using Stata V.17 (Stata Corp.).

### Replication study

#### Study population

The UK Biobank study recruited over 500 000 adults, aged 40–69 years, across 22 different sites covering England, Wales and Scotland, between 2006 and 2010.^24^ Participants completed a baseline assessment with a detailed health questionnaire and clinical measurements, which included spirometry. Between 2014 and 2020, individuals living within close proximity of an assessment site were invited for repeat assessment

#### Procedures

Participants were included in this study if they had acceptable spirometry at both baseline and follow-up. Spirometry was performed prebronchodilator (participants were not instructed to withhold their usual inhaled medications) using a calibrated Vitalograph Pneumotrac 6800. We included only those with the highest quality spirometry manoeuvres, defined as a having minimum of two spirograms with no cough, back-extrapolated volume<5% FVC (or>5% but <150 mL), reproducible FEV_1_ and FVC, and a forced expiratory time of≥6 s on the best curve (curve with highest FEV_1_ and FVC). FEF_25-75_ and FEV_3_ were derived from the raw data as previously described.^25^ For participants who attended for more than one follow-up visit, airflow obstruction was defined at its first presentation.

#### Statistical analysis

We conducted the same analysis used for the BOLD data, further adjusting for follow-up time, as this was not determined at site level. We also performed sensitivity analyses excluding those with a self-reported doctor diagnosis of asthma at baseline. This was done to make the results comparable to the postbronchodilator estimates in the BOLD study.

#### Patient and public involvement

Patients or the public were not involved in the design, or conduct, or reporting, or dissemination plans of our research.

## Results

### Main study

At baseline, 26 448 participants, across 41 sites, completed the core study questionnaire and had acceptable measurements for FEF_25-75_ and FEV_3_/FVC ratio. Eighteen study sites took part in follow-up, with 12 520 eligible participants. At follow-up, 1155 participants had died, 3658 had migrated or were unreachable, and 1237 refused to participate. Five thousand nine hundred and thirty-six participants completed the core questionnaire at follow-up, from which 1979 participants were excluded due to not performing spirometry (n=855) or poor-quality spirometry (n=1124). A total of 3957 participants with a median (IQR) follow-up time of 8.3 years (6.1–11.0) were included in the present analysis (table 1). There were fewer males than females (1733 vs 2224). Mean age ranged from 46.1 to 61.9 years across study sites. Mean BMI was lowest in Chikwawa, Malawi (21.9 kg/m²) and highest in Jamaica (30.2 kg/m²). The site with the largest proportion of never smokers was Sémé-Kpodji, Benin (99%, 150 of 152 participants), and the smallest proportion was Bergen, Norway (34%, 81 of 237 participants). At baseline, the prevalence of isolated small airways obstruction for FEF_25-75_ ranged from 0% in Tartu, Estonia and Fez, Morocco to 27.9% in Mysore, India. For FEV_3_/FVC ratio, prevalence ranged from 0% in Jamaica and Fes, Morocco to 11.6% in Reykjavik, Iceland. The prevalence of chronic airflow obstruction at baseline ranged from 4.3% in Naryn, Kyrgyzstan to 24.6% in Kashmir, India. At follow-up, the prevalence of chronic airflow obstruction was similar to baseline, ranging from 3.8% in Sémé-Kpodji, Benin to 25.0% in Kashmir, India. Mean follow-up time ranged from 4.4 years in Karachi, Pakistan to 14.7 years in Reykjavik, Iceland (table 1).

**Table 1:** Characteristics and prevalence estimates for chronic airflow obstruction (CAO) and isolated small airways obstruction (SAO) for Burden of Obstructive Lung Disease (BOLD) study participants according to participation at follow-up

BOLD centre	*n*	Baseline								Follow-up			
		Males n (%)	Age, year Mean (SD)	BMI, kg/m² Mean (SD)	Never smoke n (%)	Smoking Pack years, Mean (SD)	CAO % (SE)	Isolated SAO FEF_25-75_ % (SE)	Isolated SAO FEV_3_/FVC % (SE)	Follow-up time, years Mean (SD)	CAO % (SE)	Isolated SAO FEF_25-75_ % (SE)	Isolated SAO FEV_3_/FVC % (SE)
Benin (Sémé-Kpodji)	152	79 (52)	50.9 (8.7)	27.0 (5.5)	150 (99)	0.2 (1.5)	6.9 (2.0)	22.8 (3.9)	8.4 (2.6)	7.0 (0.2)	3.8 (1.5)	13.0 (3.2)	0
Estonia (Tartu)	139	65 (47)	61.9 (10.1)	28.2 (4.7)	82 (59)	7.2 (12.1)	9.7 (2.7)	0	9.6 (2.8)	10.6 (0.7)	14.3 (3.0)	0	2.2 (1.5)
Iceland (Reykjavik)	270	137 (51)	50.8 (8.1)	27.7 (4.6)	116 (43)	9.0 (12.5)	6.2 (1.6)	3.9 (1.6)	11.6 (2.6)	14.7 (0.5)	10.6 (2.1)	2.0 (1.0)	15.8 (2.8)
India (Mysore)	418	165 (39)	46.1 (6.6)	24.6 (3.7)	392 (94)	0.7 (3.2)	6.6 (1.3)	27.9 (2.4)	4.3 (1.1)	7.2 (0.8)	4.5 (1.1)	17.6 (2.1)	1.9 90.8)
India (Pune)	481	270 (56)	50.3 (8.5)	22.4 (3.8)	439 (91)	0.5 (2.5)	5.4 (1.1)	19.8 (1.9)	5.4 (1.1)	11.0 (0.4)	5.8 (1.3)	12.3 (1.8)	1.1 (0.6)
India (Kashmir)	27	17 (63)	52.0 (9.5)	22.2 (3.3)	24 (89)	0.8 (3.0)	24.6 (8.7)	5.6 (5.4)	5.2 (5.1)	8.5 (0.4)	25.0 (8.4)	0	0
Jamaica	26	15 (58)	52.5 (8.2)	30.2 (8.3)	19 (73)	1.4 (3.6)	7.9 (5.3)	7.6 (5.3)	0	5.5 (0.2)	4.0 (3.9)	4.4 (4.3)	0
Kyrgyzstan (Chui)	333	92 (28)	51.3 (7.6)	28.6 (5.5)	248 (75)	5.7 (14.7)	10.2 (1.7)	8.5 (1.7)	5.4 (1.4)	6.2 (0.2)	11.3 (1.8)	6.2 (1.5)	6.0 (1.6)
Kyrgyzstan (Naryn)	296	110 (37)	50.7 (7.6)	27.0 (4.9)	231 (78)	3.9 (10.1)	4.3 (1.2)	5.7 (1.5)	9.3 (1.8)	6.2 (0.1)	5.7 (1.4)	6.5 (1.6)	5.8 (1.6)
Malawi (Chikwawa)	260	117 (45)	53.1 (10.0)	21.9 (3.8)	190 (73)	1.7 (4.7)	8.9 (1.8)	15.0 (2.5)	6.5 (1.7)	4.8 (0.4)	13.4 (2.1)	16.5 (3.0)	15.4 (2.8)
Morocco (Fes)	18	13 (72)	49.8 (5.3)	26.3 (4.8)	9 (50)	15.1 (18.6)	4.7 (4.6)	0	0	10.5 (0.3)	4.7 (4.6)	0	0
Nigeria (Ife)	400	112 (28)	54.7 (11.3)	25.8 (5.6)	372 (93)	0.3 (1.9)	7.7 (1.4)	18.1 (2.2)	9.9 (1.70	8.3 (0.2)	4.8 (1.3)	10.9 (1.8)	1.1 (0.7)
Norway (Bergen)	237	117 (49)	53.8 (8.4)	26.2 (4.0)	81 (34)	11.6 (14.0)	9.5 (1.9)	3.2 (1.4)	10.4 (2.4)	9.3 (5.6)	11.5 (2.2)	1.6 (0.1)	6.7 (1.8)
Pakistan (Karachi)	207	85 (41)	49.9 (8.5)	26.9 (5.4)	164 (79)	3.9 (11.7)	6.7 (1.8)	22.4 (3.2)	6.7 (1.9)	4.4 (0.4)	4.5 (1.5)	19.7 (3.2)	1.2 (0.8)
Philippines (Nampicuan-Talugtug)	276	123 (45)	51.0 (8.4)	22.0 (4.2)	151 (55)	8.2 (12.4)	8.2 (1.7)	14.9 (2.4)	5.9 (1.6)	10.8 (0.3)	12.0 (2.0)	11.3 (2.2)	3.7 (1.4)
Sudan (Khartoum)	36	22 (61)	51.2 (9.4)	27.5 (6.1)	21 (58)	6.3 (9.7)	4.4 (4.4)	12.7 (8.4)	6.1 (5.9)	7.7 (0.4)	5.6 (3.8)	20.6 (10.7)	7.5 (7.2)
Sweden (Uppsala)	219	120 (55)	54.7 (8.1)	26.7 (3.8)	93 (42)	9.1 (13.9)	5.9 (1.6)	4.1 (1.6)	6.2 (2.0)	13.3 (0.6)	4.5 (1.4)	0.7 (0.7)	3.7 (1.5)
Tunisia (Sousse)	162	74 (46)	52.4 (8.6)	29.8 (5.4)	105 (64)	10.6 (18.3)	7.8 (2.4)	4.3 (1.7)	2.7 (1.4)	10.4 (0.5)	12.1 (2.8)	8.7 (2.7)	4.6 (2.0)

Smoking pack years were calculated by number of cigarettes smoked per day divided by 20 and multiplied by years of smoking. CAO: chronic airflow obstruction, identified if the postbronchodilator forced expiratory volume in 1 s as a ratio of the forced vital capacity (FEV_1_/FVC ratio) was less than the lower limit of normal (LLN) given age, sex, and height using European American reference equations from the National Health and Nutrition Examination Survey.^17 18^ Isolated SAO identified if the prebronchodilator mean forced expiratory flow rate between 25% and 75% of the forced vital capacity (FEF_25-75_) or the forced expiratory volume in 3 s as a ratio of the forced vital capacity (FEV_3_/FVC) were less than the LLN. All prevalence estimates were calculated using inverse probability weights to account for missing data and as such are reported as % (SE).

Table 2 shows the characteristics of BOLD study participants with isolated small airways obstruction at baseline. Participants with isolated small airways obstruction for FEF_25-75_, were on average younger, more likely to be female, and to be never smokers than those with isolated small airways obstruction for FEV_3_/FVC ratio. They also had a smaller smoking pack year history, higher FEV_1_/FVC ratio and lower FVC.

**Table 2:** Characteristics of those with isolated small airways obstruction (SAO) at baseline in the BOLD and UK Biobank studies according to participation at follow-up

	BOLD Study		UK Biobank study	
	Isolated SAO FEF_25-75_<LLN	Isolated SAO FEV_3_/FVC<LLN	Isolated SAO FEF_25-75_<LLN	Isolated SAO FEV_3_/FVC<LLN
N	448	233	549	162
Age years, Mean (SD)	47.7 (6.5)	52.6 (8.6)	50.8 (6.6)	53.2
Females, n (%)	290 (69)	101 (46)	413 (75)	107 (66)
BMI kg/m², mean (SD)	25.1 (5.4)	26.0 (5.0)	27.0 (5.4)	26.5 (4.8)
Never smoked, n (%)	352 (84)	144 (65)	326 (60)	96 (59)
Current smoker, n (%)	44 (11)	40 (18)	67 (12)	8 (5)
Former smoker, n (%)	23 (6)	37 (17)	156 (28)	58 (36)
Smoking pack years, mean (SD)	2.3 (7.7)	6.5 (13.1)	7.0 (13.9)	6.0 (12.7)
FEV_1_/FVC %, mean (SD)	74.5 (3.8)	72.5 (5.3)	72.4 (2.8)	75.7 (4.0)
FVC L, mean (SD)	2.5 (0.7)	3.4 (1.1)	2.8 (0.7)	3.6 (0.9)
FEF_25-75_ L/min, mean (SD)	1.3 (0.4)	1.6 (0.6)	1.4 (0.4)	2.1 (0.7)
FEV_3_/FVC %, mean (SD)	89.4 (3.9)	84.1 (3.1)	90.4 (2.7)	83.9 (2.9)

Isolated SAO identified if the prebronchodilator FEF_25-75_ or FEV_3_/FVC were less than the LLN in the presence of an FEV_1_/FVC greater than or equal to the LLN using European American reference equations from the National Health and Nutrition Examination Survey.^17 18^

BMI, body mass index; FEF_25-75_, mean forced expiratory flow rate between 25% and 75% of the forced vital capacity; FEV_1_/FVC, forced expiratory volume in 1 s as a ratio of the forced vital capacity; FEV_3_/FVC, forced expiratory volume in 3 s as a ratio of the forced vital capacity; FVC, forced vital capacity; Smoking pack years, calculated by number of cigarettes smoked per day divided by 20 and multiplied by years of smoking.

**Figure 1:**
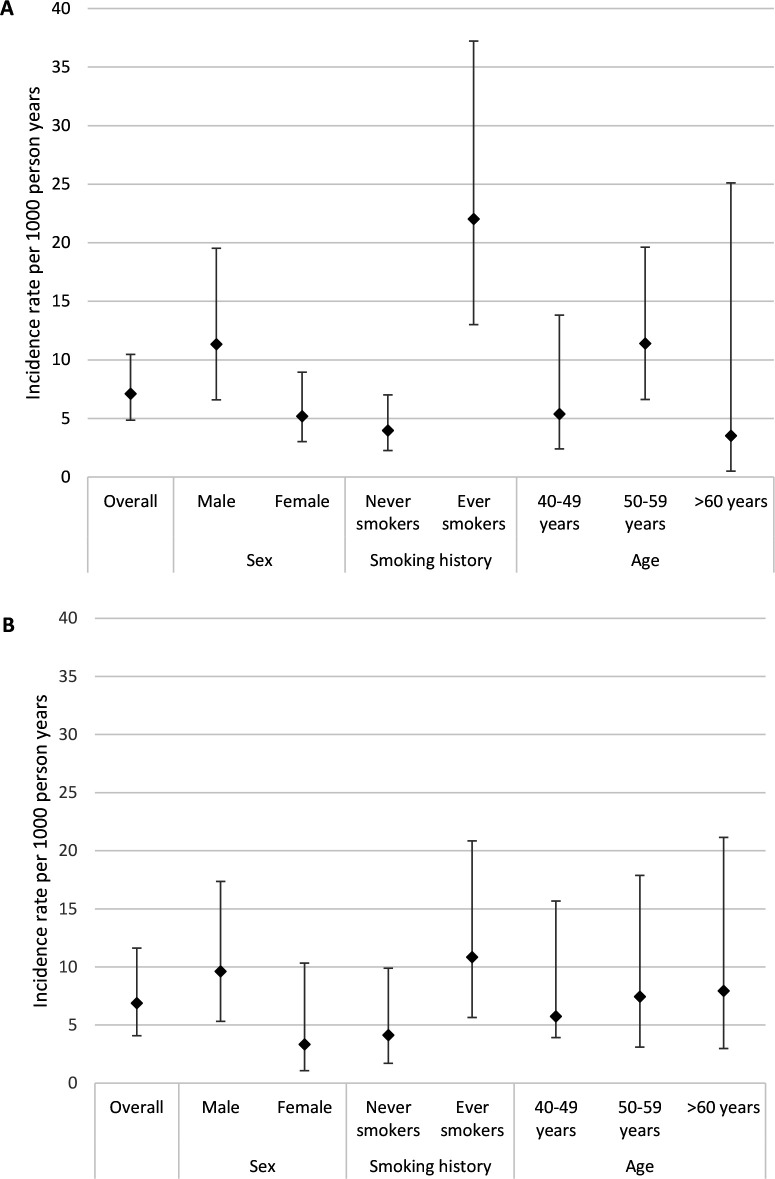
Incidence rate per 1000 person years for progression from isolated small airways obstruction to chronic airflow obstruction for A) FEF_25-75_ and B) FEV_3_/FVC ratio.

Of those with isolated small airways obstruction for FEF_25-75_, 26 of 448 (6%) progressed to chronic airflow obstruction at follow-up. While for FEV_3_/FVC ratio, 14 of 233 (6%) progressed to chronic airflow obstruction. In participants with no evidence of any airway obstruction at baseline, 72 of 2545 (3%) progressed to chronic airflow obstruction. The incidence rates for progression to chronic airflow obstruction were 7.1/1000 person years (95% CI 4.9 to 10.5) for FEF_25-75_ less than the LLN and 6.9/1000 (95% CI 4.1 to 11.6) for FEV_3_/FVC ratio less than the LLN. In individuals with no evidence of airflow obstruction at baseline (ie, FEF_25-75_, FEV_3_/FVC, and FEV_1_/FVC greater than or equal to the LLN), incidence of progression to chronic airflow obstruction was 3.2/1000 (95% CI 2.6 to 4.1). For both parameters, incidence rates were higher in males than females and in ever smokers compared with never smokers (figure 1).

When stratifying by WHO region, incidence rates for progression to chronic airflow obstruction for FEF_25-75_, ranged from 2.4/1000 (95% CI 0.6 to 9.6) in the African region to 23.0/1000 (95% CI 12.7 to 41.5) in the European region. While for FEV_3_/FVC ratio, incidence ranged from 0.0/1000 in the Eastern Mediterranean region to 7.6/1000 (95% CI 3.6 to 15.9) in the European region (online supplemental etable 1, appendix p2).

10.1136/bmjresp-2023-002056.supp1 Supplementary data

Tables 3 and 4 display the results of the mixed effects regression analyses. At baseline, isolated small airways obstruction for FEF_25-75_ was associated with a lower FEV_1_/FVC ratio (β: −4.16, 95% CI −5.70 to –2.63), and significantly increased odds of chronic airflow obstruction at follow-up (OR: 2.95, 95% CI 1.02 to 8.54). Similarly, isolated small airways obstruction for FEV_3_/FVC ratio was associated with a lower FEV_1_/FVC ratio (β: −2.87 95% CI −4.27 to –1.47) and chronic airflow obstruction at follow-up (OR: 1.94, 95% CI 1.05 to 3.62). After excluding ever smokers from the analysis, isolated small airways obstruction for FEF_25-75_ (β: −3.41, 95% CI −4.88 to –1.95) and FEV_3_/FVC ratio (β: −2.58, 95% CI −3.97 to –1.21) were associated with a lower FEV_1_/FVC ratio but not chronic airflow obstruction. When stratifying by follow-up time, there were no significant differences in the association with FEV_1_/FVC ratio for either parameter. Isolated small airways obstruction for both FEF_25-75_ and FEV_3_/FVC ratio were associated with a significantly lower FEV_1_/FVC ratio at follow-up in both males and females. However, only in males was there an association with subsequent chronic airflow obstruction (tables 3 and 4). Of note, 44% of male participants reported a smoking history, compared with just 14% of females. The results for postbronchodilator FEF_25-75_ and FEV_3_/FVC ratio were not materially different from prebronchodilator (online supplemental table 2 and 3, appendix p3).

**Table 3:** Association between baseline isolated small airways obstruction (SAO) and chronic airflow obstruction (CAO) at follow-up for FEF_25-75_

	Total n	Isolated SAO (baseline) n	CAO (follow-up) n	OR (95% CI)	P value	β coefficient (95% CI)^*^	P value
Overall model	3040	448	26	2.95 (1.02 to 8.54)	0.046	−4.16 (−5.70 to 2.63)	<0.0001
Follow-up time<5 years	360	60	4	2.66 (0.66 to 10.69)	0.168	−5.07 (−5.72 to 4.43)	<0.0001
Follow-up time≥5 years	2680	388	22	3.41 (0.81 to 14.34)	0.093	−4.15 (−6.00 to 2.31)	<0.0001
Stratified by sex							
Male	1329	138	13	4.62 (1.09 to 19.61)	0.038	−5.19 (−7.14 to 3.24)	<0.0001
Female	1711	310	13	2.32 (0.45 to 11.85)	0.311	−3.13 (−4.69 to 1.58)	<0.0001
Never smoked	2300	378	12	1.45 (0.38 to 5.47)	0.587	−3.41 (−4.88 to 1.95)	<0.0001

Linear associations between prebronchodilator isolated SAO for FEF_25-75_ at baseline and follow-up postbronchodilator FEV_1_/FVC ratio were estimated using mixed effects linear regression models.

*Negative regression coefficient indicates a reduction in FEV_1_/FVC ratio (ie, worsened lung function). Associations between isolated SAO at baseline and progression to CAO were estimated using mixed effects logistic regression models. Models were adjusted for sex, age, BMI, smoking status and smoking pack years. As we expected associations to vary by study site, we fitted a random slope model to average the associations across study sites. Isolated SAO was identified if the prebronchodilator mean forced expiratory flow rate between 25% and 75% of the forced vital capacity (FEF_25-75_) was below the lower limit of normal (<LLN) and the prebronchodilator forced expiratory volume in 1 s as a ratio of the forced vital capacity (FEV_1_/FVC ratio) was equal to or above the lower limit of normal (≥LLN) at baseline. CAO was diagnosed if the postbronchodilator (200 μg salbutamol) FEV_1_/FVC ratio was <LLN at follow up. Lower limit of normal calculated using reference equations from the NHANES III study population^17 18^. Total n= those without CAO at baseline who had a measurement for FEF_25-75._

**Table 4:** Association between baseline isolated small airways obstruction (SAO) and chronic airflow obstruction (CAO) at follow-up for FEV_3_/FVC ratio.

	Total n	Isolated SAO (baseline) n	CAO (follow-up) n	OR (95% CI)	P value	β coefficient (95% CI)^*^	P value
Overall model	3140	233	14	1.94 (1.05 to 3.62)	0.035	−2.87 (−4.27 to 1.47)	<0.0001
Follow-up time<5 years	377	33	0	–	–	−3.79 (−7.33 to 0.25)	0.036
Follow-up time≥5 years	2763	200	14	2.29 (1.19 to 4.40)	0.013	−2.87 (−4.33 to 1.40)	<0.0001
Stratified by sex							
Male	1369	127	11	2.44 (1.19 to 4.98)	0.014	−3.94 (−5.19 to 2.68)	<0.0001
Female	1771	106	3	0.55 (0.03 to 10.82)	0.698	−1.65 (−3.32 to 0.02)	0.053
Never smoked	2379	151	5	1.36 (0.53 to 3.50)	0.521	−2.58 (−3.97 to 1.21)	<0.0001

Linear associations between prebronchodilator isolated small airways obstruction (SAO) for FEV_3_/FVC ratio at baseline and follow-up post-bronchodilator FEV_1_/FVC ratio were estimated using mixed effects linear regression models.

*Negative regression coefficient indicates a reduction in FEV_1_/FVC ratio (ie, worsened lung function). Associations between isolated SAO at baseline and progression to CAO were estimated using mixed effects logistic regression models. Models were adjusted for sex, age, BMI, smoking status, and smoking pack years. As we expected associations to vary by study site, we fitted a random slope model to average the associations across study sites. Isolated SAO was identified if the prebronchodilator forced expiratory volume in 3 s as a ratio of the forced vital capacity (FEV_3_/FVC ratio) was below the lower limit of normal (<LLN) and the prebronchodilator forced expiratory volume in 1 s as a ratio of the forced vital capacity (FEV_1_/FVC ratio) was equal to or above the lower limit of normal (≥LLN) at baseline. CAO was diagnosed if the postbronchodilator (200 µg salbutamol) FEV_1_/FVC ratio was <LLN at follow up. Lower limit of normal calculated using reference equations from the US National Health and Nutrition Examination Survey III study population^17 18^. Total n= those without CAO at baseline who had a measurement for FEV_3_/FVC ratio_._

**Figure 2:**
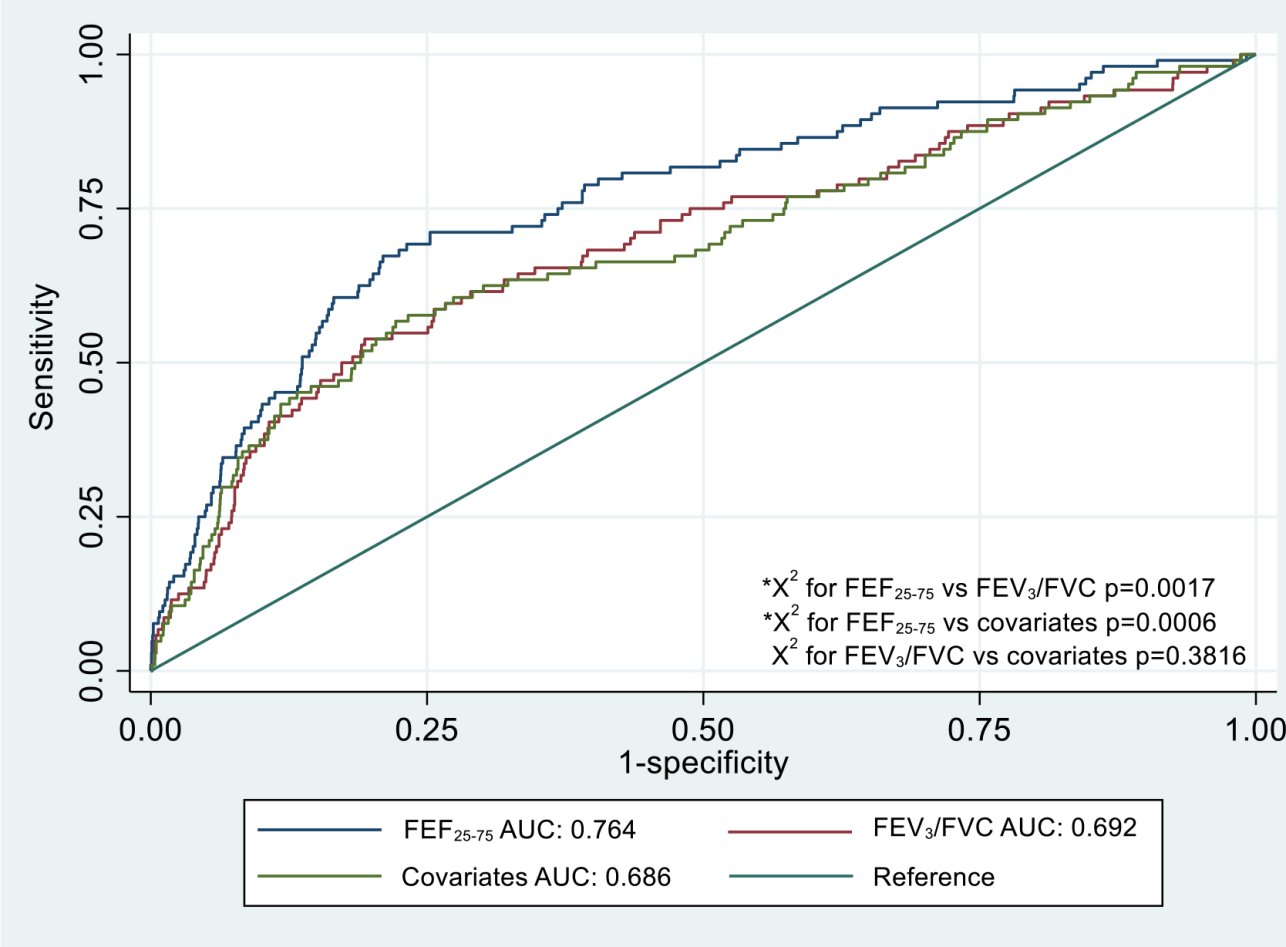
Receiver operator characteristic curve and area under the curve (AUC) comparing ability of FEF_25-75_ and FEV_3_/FVC ratio to a model containing age, sex, BMI and smoking history alone to discriminate future chronic airflow obstruction. *P-value less than 0.05 indicates significant difference between models according to X^2^ test.

The AUC to discriminate progression to chronic airflow obstruction was 0.764 for FEF_25-75_ and 0.692 for FEV_3_/FVC ratio (figure 2). There was a significant difference in the AUC between the two parameters (p=0.0017). When compared with the AUC for a model containing just age, sex, BMI and smoking history (AUC=0.686), FEF_25-75_ significantly improved discrimination (p=0.0006), while FEV_3_/FVC ratio did not (p=0.3816). The Brier scores assessing the predictive accuracy of the parameters was 0.0322 for FEF_25-75_ and 0.0320 for FEV_3_/FVC ratio, indicating good predictive accuracy.

### Replication study

Two hundred and fifty-two thousand five hundred and sixty participants had high-quality spirometry at baseline. Of these, 26 512 did not have airflow obstruction at baseline, had high quality spirometry at follow-up and were included in this analysis. Forty-two per cent were male, with a mean age of 55.5 years. Mean BMI was 26.7 kg/m² and 60% were never smokers. At baseline, 549 (2%) participants had isolated small airways obstruction for FEF_25-75_ and 162 (1%) for FEV_3_/FVC ratio. Median (IQR) follow-up time was 8.0 years (IQR: 5.0–10.0). Like the BOLD study participants, UK Biobank participants with isolated small airways obstruction for FEF_25-75_ were generally younger, more likely to be females, and have a lower FVC than those with isolated small airways obstruction for FEV_3_/FVC ratio. Interestingly, unlike in the BOLD study, those with isolated small airways obstruction for FEF_25-75_ had the lower FEV_1_/FVC ratio. When comparing across cohorts, the characteristics of those with isolated small airways obstruction were similar. The exceptions being that there were less never smokers and more former smokers in the UK Biobank study, as well as a larger smoking pack year history (table 2).

Of those with normal lung function at baseline, 1877 of 25 832 (7%) progressed to airflow obstruction (9.82/1000 person years, 95% CI 9.38 to 10.27). For FEF_25-75_, 116 of 549 (21%) progressed to airflow obstruction (28.77/1000 person years, 95% CI 23.98 to 34.91), and for FEV_3_/FVC ratio, 17 of 162 (10%) progressed to airflow obstruction (14.22/1000 person years, 95% CI 8.84 to 22.88).

Isolated small airways obstruction for FEF_25-75_ was associated with a lower FEV_1_/FVC ratio (β: −4.45, 95% CI −5.05 to –3.85) and greater odds of progression to airflow obstruction at follow-up (OR: 3.79, 95% CI 3.10 to 4.71). This association was seen in both males and females, and those who had never smoked (online supplemental etable 4, appendix). There was no association between isolated small airways obstruction for FEV_3_/FVC ratio and the FEV_1_/FVC ratio at follow-up (online supplemental etable 5, appendix). When stratifying by follow-up time, there were no significant differences in the associations for either parameter. After excluding those with a self-reported doctor diagnosis of asthma (online supplemental etable 6, appendix), associations with isolated small airways obstruction for FEF_25-75_ did not materially change. However, those with isolated small airways obstruction for FEV_3_/FVC ratio had a lower FEV_1_/FVC ratio (β: −2.02, 95% CI −3.61 to –0.43) and greater odds of progressing to airflow obstruction (OR: 2.65, 95% CI 1.45 to 4.82).

The AUC for FEF_25-75_ was 0.692, which improved discrimination compared with FEV_3_/FVC ratio (AUC: 0.629) (online supplemental figure 1, appendix). For both parameters, isolated small airways obstruction improved discrimination compared with a model containing age, sex, BMI and smoking history. The brier scores were 0.096 and 0.1042 for FEF_25-75_ and FEV_3_/FVC ratio, respectively. Restricting the analyses to those without a self-reported history of asthma did not improve discrimination (online supplemental efigure 2, appendix).

## Discussion

To the best of our knowledge, this is the first general-population study to investigate whether isolated small airways obstruction is associated with progression to chronic airflow obstruction over time. Our study shows that isolated small airways obstruction is associated with having a lower FEV_1_/FVC ratio and increased odds of chronic airflow obstruction later in life. In addition, we found that isolated small airways obstruction measured using FEF_25-75_ was a better predictor of future obstruction than the FEV_3_/FVC ratio. We successfully replicated these findings using data from the UK Biobank study.

In the present study, when compared with those with no evidence of airflow obstruction at baseline, isolated small airways obstruction for FEF_25-75_ was associated with a 4% lower FEV_1_/FVC ratio and three times greater odds of chronic airflow obstruction at follow-up. We found similar in the UK Biobank study. Only one study has previously investigated this association. Kwon *et al*^12^ showed, in a hospital-based population of South Korean adults, that an isolated abnormality in FEF_25-75_ was associated with increased risk of airflow obstruction over a 10-year period. They also showed that risk of progression was higher for those with a smoking history. Unlike our study, Kwon *et al*^12^ used the fixed ratio of 0.70 for FEV_1_/FVC to define chronic airflow obstruction. The limitations of using a fixed ratio in general populations are well known and relate to overestimation of incidence. For this reason, its use is no longer recommended by the American Thoracic Society and European Respiratory Society.^26^ Our study adds to their findings by showing that the same association is seen when using the LLN to define abnormality in general populations.

At baseline, isolated small airways obstruction for FEV_3_/FVC ratio was associated with a 3% lower FEV_1_/FVC ratio and two times greater odds of progression to chronic airflow obstruction at follow-up. We replicated these findings in UK biobank participants but only after exclusion of those with a self-reported history of asthma. No previous studies have investigated this association. However, Dilektasli *et al*^9^ reported that in ever smokers from the COPDGene study, when the FEV_1_/FVC ratio was greater than 0.70, FEV_3_/FVC ratio less than the LLN was associated with increased emphysema and gas trapping on CT imaging, supporting our finding that an abnormal FEV_3_/FVC ratio is a precursor to future obstructive lung disease. In addition, studies in ever smokers have shown that having a FEV_3_/FEV_6_ ratio less than the LLN is also associated with increased risk of chronic airflow obstruction.^8 27^ The FEV_3_/FVC ratio and FEV_3_/FEV_6_ ratio are highly correlated and shown to give very similar prevalence estimates for isolated small airways obstruction.^28^ The rationale behind using the FEV_6_ in place of the FVC is uncertain providing spirometry has been performed correctly.

Interestingly, in the BOLD study, we found that isolated small airways obstruction was associated with progression to chronic airflow obstruction only in males. However, it is not clear if this is a genuine interaction, as in contrast with the logistic regression, the results of the linear regression showed that regardless of sex, those with isolated small airways obstruction had a lower FEV_1_/FVC ratio at follow-up. A potential explanation is the far smaller proportion of females that reported a smoking history compared with males; an important difference considering smoking is the strongest risk factor for chronic airflow obstruction.^19 29^ In our replication study, females with isolated small airways obstruction had a lower FEV_1_/FVC ratio and greater odds of progression to airflow obstruction at follow-up. This makes it likely that despite the association being stronger in males, universally, isolated small airways obstruction is a good predictor of future airflow obstruction.

Our finding that in never smokers, isolated small airways obstruction was associated with having a significantly lower FEV_1_/FVC ratio at follow-up is novel and was successfully replicated in participants of the UK Biobank study. It is well known that cigarette smoke damages the small airways, eventually leading to chronic airflow obstruction.^19^ We have previously shown that risk factors for isolated small airways obstruction also include occupational exposures to dust, previous TB diagnosis, low education level and family history of COPD.^29^ The causal pathways in never smokers are less clear and deserve further research, especially on the impact of intrauterine exposures, childhood growth, and ambient and indoor air pollution.

No previous studies have reported incidence rates for progression of isolated small airways obstruction to chronic airflow obstruction. In the BOLD study, we found that incidence of progression was similar for both parameters. It was higher in males compared with females, and ever smokers compared with never smokers. We found considerable variation in incidence rates across WHO regions. Despite this, incidence of progression to chronic airflow obstruction was generally highest in the European region. The incidence rates for progression from isolated small airways obstruction to airflow obstruction were significantly higher in our replication study. However, they were similar to BOLD sites in the European region. This finding is likely related to tobacco smoking, as in the European region of the BOLD study and the UK Biobank study, 43% and 40% of participants, respectively, were smokers.

Due to lack of agreement as to which spirometry parameters best reflects changes within the small airways, we used both FEF_25-75_ and FEV_3_/FVC. Both parameters have been shown to correlate with functional small airways disease on CT imaging,^7 9 10^ and we found that when less than the LLN, both are also associated with progression to chronic airflow obstruction. When we calculated the AUC of the parameters, FEF_25-75_ was significantly better at discriminating future chronic airflow obstruction than the FEV_3_/FVC ratio. In addition, when compared with a model containing age, sex, BMI and smoking history alone, only FEF_25-75_ significantly improved discrimination. Predictive accuracy using the Brier score was good for both parameters. We found a similar pattern in the UK Biobank study; however, overall discriminative ability was weaker. This could be explained by the optimisation of the statistical model for participants of the BOLD study, meaning there could be additional covariates that influence discriminative ability in the UK Biobank study that are not significant or are unmeasured in the BOLD study. Despite this, our findings suggest that while both FEF_25-75_ and FEV_3_/FVC ratio can be used to identify those at risk of chronic airflow obstruction, FEF_25-75_ performs the best.

Our study has strengths, including a wide geographical coverage, samples that are representative of the general population, and quality assured spirometry. We also compared the predictive ability of two different spirometry parameters. Our decision to prioritise prebronchodilator spirometry to define isolated small airways obstruction means at-risk individuals can be identified without the need for postbronchodilator spirometry, which is time consuming, costly and not widely available in low resource settings. There are also limitations. First, in a previous publication, we found that there was minimal difference between the FVC and forced expiratory volume in six seconds (FEV_6_) in UK Biobank partiscipants.^25^ This suggests that the FVC may be underestimated, which would falsely increase the FEF_25-75_ and FEV_3_/FVC ratio. As a result, prevalence if isolated small airways obstruction would be underestimated and the power to find an association reduced, particularly for the FEV_3_/FVC ratio. Second, due to the COVID-19 pandemic, the BOLD study had considerable loss to follow-up at some sites. However, we used inverse probability weighting in our analyses to account for this.

## Conclusion

People with isolated small airways obstruction, particularly when measured using FEF_25-75_, are more likely to develop chronic airflow obstruction over time. As chronic airflow obstruction is a key component of a COPD diagnosis, our findings have implications for early detection and prevention of disease.

## Data Availability

Data are available upon reasonable request. Deidentified participant data and questionnaires of the BOLD study may be shared, after publication, on a collaborative basis upon reasonable request made to Dr Amaral (a.amaral@imperial.ac.uk). Requesting researchers will be required to submit an analysis plan.
