# Supplementary material for: Isolated small airways obstruction predicts future chronic airflow obstruction: a multinational longitudinal study
Source: BMJ Open Respir Res. 2023 Nov 20;10(1):e002056. doi: 10.1136/bmjresp-2023-002056 (PMC10660204; doi:10.1136/bmjresp-2023-002056)
Supplement: Supplementary data [file bmjresp-2023-002056supp001.pdf]

Supplemental appendix

Title: Isolated small airways obstruction predicts future chronic airflow obstruction: A multinational longitudinal study

Contents

Page 2…… eTable 1. Incidence rates per 1000 person years for progression from isolated small airways obstruction at baseline to chronic airflow obstruction at follow-up according to WHO region and study site.

Page 3…… eTable 2. Association between post-bronchodilator isolated small airways obstruction at baseline and chronic airflow obstruction at follow-up for FEF_{25-75} and eTable 3. Association between post-bronchodilator isolated small airways obstruction at baseline and chronic airflow obstruction at follow-up for FEV_3/FVC

Page 4…… eTable 4. Association between baseline isolated small airways obstruction and airflow obstruction at follow-up in the UK Biobank for FEF_{25-75}. eTable 5**.** Association between baseline isolated small airways obstruction and airflow obstruction at follow-up in the UK Biobank for FEV_3/FVC.

Page 5…… eFigure 1. Receiver operator characteristic curve and area under the curve (AUC) comparing ability of FEF_{25-75} and FEV_3/FVC ratio to a model containing age, sex, BMI and smoking history alone to discriminate future airflow obstruction in UK Biobank participants.

Page 6…… eTable 6. Association between baseline isolated small airways obstruction and airflow obstruction at follow-up in the UK Biobank excluding those with asthma**.**

Page 7…… eFigure 2. Receiver operator characteristic curve and area under the curve (AUC) comparing ability of FEF_{25-75} and FEV_3/FVC ratio to discriminate future airflow obstruction in UK Biobank participants with no self-reported asthma at baseline.

.

eTable 1. Incidence rates per 1000 person years for progression from isolated small airways obstruction at baseline to chronic airflow obstruction at follow-up according to WHO region and study site.

World region/BOLD Centre	FEF_{25-75}				FEV_3/FVC			
	Baseline isolated SAO FEF_{25-75} *n=*	CAO at follow-up *n=*	Person years	Incidence rate per 1000 person-years (95%CI)	Baseline isolated SAO FEV_3/FVC *n=*	CAO at follow-up *n=*	Person years	Incidence rate per 1000 person-years (95%CI)
**European**	**58**	**11**	**478.7**	**23.0 (12.7-41.5)**	**97**	**7**	**924.6**	**7.6 (3.6-15.9)**
Estonia (Tartu)	0	0	0.0	0.0	11	1	119.5	8.4 (1.18-59.4)
Iceland (Reykjavik)	6	3	88.9	33.7 (10.9-104.6)	19	3	281.0	10.7 (3.4-33.1)
Kyrgyzstan (Chui)	24	4	149.5	26.8 (10.0-71.3)	15	1	92.76	10.8 (1.5-76.5)
Kyrgyzstan (Naryn)	15	1	95.6	10.8 (1.5-76.7)	24	1	147.6	6.8 (1.0-48.1)
Norway (Bergen)	6	0	65.4	0.0	19	0	172.1	0.0
Sweden (Uppsala)	6	3	82.3	36.5 (11.7-113.0)	9	1	111.6	9.0 (1.3-64.0)
**Eastern Mediterranean**	**48**	**4**	**256.6**	**15.6 (5.9, 41.5)**	**21**	**0**	**117.9**	**0.0**
Morocco (Fes)	0	0	0.0	0.0	0	0	0.0	0.0
Pakistan (Karachi)	38	2	163.0	12.3 (3.1-49.1)	16	0	68.0	
Sudan (Khartoum)	4	0	30.3	0.0	1	0	7.1	0.0
Tunisia (Sousse)	6	2	63.3	31.6 (7.9-126.4)	4	0	42.8	0.0
**South-East Asian**	**190**	**6**	**1689**	**3.6 (1.6-7.9)**	**44**	**3**	**424.7**	**7.1 (2.28-21.9)**
India (Mysore)	101	1	720.0	1.39 (0.2-9.9)	17	1	130.1	7.69 (1.08-54.6)
India (Pune)	88	5	961.0	5.2 (2.2-12.5)	26	2	286.1	7.0 (1.8 (1.7-8.0)
India (Kashmir)	1	0	8.5	0.0	1	0	8.5	0.0
**African**	**116**	**2**	**831.4**	**2.4 (0.6-9.6)**	**57**	**2**	**411.6**	**4.9 (1.2-19.4)**
Benin (Sémé-Kpodji)	27	0	188.7	0.0	11	0	77.0	0.0
Malawi (Chikwawa)	31	2	150.1	13.4 (3.4-53.3)	14	1	68.8	14.5 (2.5-103.2
Nigeria (Ife)	58	0	492.6	0.0	32	1	265.8	3.76 (0.53-26.7)
**Western Pacific**	**35**	**3**	**380.9**	**7.9 (2.5-24.4)**	**14**	**2**	**154.0**	**13.0 (3.24-51.9)**
Philippines (Nampicuan-Talugtug)	35	3	380.9	7.9 (2.5-24.4)	14	2	154.0	13.0 (3.24-51.9)
**Americas**	**2**	**0**	**10.7**	**0.0**	**0**	**0**	**0.0**	**0.0**
Jamaica (Kingston)	2	0	10.7	0.0	0	0	0.0	0.0

Incidence rates reported per 1000 person years with 95% confidence interval. FEF_{25-75}: Mean forced expiratory flow rate between 25% and 75% of the forced vital capacity. FEV_3/FVC ratio: Forced expiratory volume in three seconds as a ratio of the forced vital capacity. Isolated reductions in FEF_{25-75} and FEV_3/FVC ratio identified if pre-bronchodilator measurment was less than the lower limit of normal with a pre-bronchodilator FEV_1/FVC equal to or above the lower limit of normal. Chronic airflow obstruction at follow-up defined as post-bronchodilator FEV_1/FVC ratio less than the lower limit of normal. Limits of normal calculated using European American reference equations from the National Health and Nutrition Examination Survey[17,18]

eTable 2. Association between post-bronchodilator isolated small airways obstruction at baseline and chronic airflow obstruction at follow-up for $FEF_{25\text{-}75}$ in the BOLD study

	**Total** *n*	**Isolated SAO (baseline)** *n*	**CAO (follow-up)** *n*	**OR (95%CI)**	**p-value**	**β coefficient (95%CI)***	**p-value**
**Overall model**	3324	332	28	3.67 (1.97, 6.83)	<0.0001	-4.14 (-5.69, -2.60)	<0.0001
**Stratified by sex**							
Male	1431	109	16	6.14 (2.99, 12.63)	<0.0001	-5.82 (-7.96, -3.69)	<0.0001
Female	1893	223	12	2.31 (0.90, 5.90)	0.080	-3.12 (-4.82, -1.42)	<0.0001
**Never smoked**	2513	286	17	3.34 (1.64, 6.79)	0.001	-4.16 (-5.87, -2.46)	<0.0001

*Linear associations between an isolated reduction in post-bronchodilator $FEF_{25\text{-}75}$ at baseline and follow-up post-bronchodilator FEV_1/FVC ratio were estimated using mixed effects linear regression models. *Negative regression coefficient indicates a reduction in FEV_1/FVC ratio (ie, worsened lung function). Associations between an isolated reduction in $FEF_{25\text{-}75}$ at baseline and progression to chronic airflow obstruction were estimated using mixed effects logistic regression models. Models were adjusted for sex, age, BMI, smoking status, and smoking pack years. As we expected associations to vary by study site, we fitted a random slope model to average the associations across study sites. Isolated reduction in $FEF_{25\text{-}75}$ identified if the post-bronchodilator mean forced expiratory flow rate between 25% and 75% of the forced vital capacity ($FEF_{25\text{-}75}$) was below the lower limit of normal (<LLN) and the post-bronchodilator forced expiratory volume in 1 second as a ratio of the forced vital capacity (FEV_1/FVC ratio) was equal to or above the lower limit of normal (≥LLN) at baseline. Chronic airflow obstruction was diagnosed if the post-bronchodilator (200mcg salbutamol) FEV_1/FVC ratio was <LLN at follow up. Lower limit of normal calculated using reference equations from the NHANES III study population[17,18]. Total n= those without chronic airflow obstruction at baseline who had a measurement for $FEF_{25\text{-}75}$.*

eTable 3. Association between post-bronchodilator isolated small airways obstruction at baseline and chronic airflow obstruction at follow-up for FEV_3/FVC in the BOLD study

	**Total** *n*	**Isolated SAO (baseline)** *n*	**CAO (follow-up)** *n*	**OR (95%CI)**	**p-value**	**β coefficient (95%CI)***	**p-value**
**Overall model**	3384	135	15	2.22 (1.23, 4.00)	0.008	-3.87 (-6.16, -1.59)	0.001
**Stratified by sex**							
Male	1451	80	13	2.57 (1.18, 5.66)	0.018	-5.05 (-7.42, -2.68)	<0.0001
Female	1933	55	2	0.76 (0.07, 8.09)	0.817	-0.87 (-2.76, 1.00)	0.360
**Never smoked**	2562	81	6	1.81 (0.46, 7.17)	0.394	-4.42 (-7.23, -1.61)	0.002

*Linear associations between an isolated reduction in post-bronchodilator FEV_3/FVC ratio at baseline and follow-up post-bronchodilator FEV_1/FVC ratio were estimated using mixed effects linear regression models. *Negative regression coefficient indicates a reduction in FEV_1/FVC ratio (ie, worsened lung function). Associations between an isolated reduction in FEV_3/FVC ratio at baseline and progression to chronic airflow obstruction were estimated using mixed effects logistic regression models. Models were adjusted for sex, age, BMI, smoking status, and smoking pack years. As we expected associations to vary by study site, we fitted a random slope model to average the associations across study sites. Isolated reduction in FEV_3/FVC ratio identified if the post-bronchodilator forced expiratory volume in 3 seconds as a ratio of the forced vital capacity (FEV_3/FVC ratio) was below the lower limit of normal (<LLN) and the post-bronchodilator forced expiratory volume in 1 second as a ratio of the forced vital capacity (FEV_1/FVC ratio) was equal to or above the lower limit of normal (≥LLN) at baseline. Chronic airflow obstruction was diagnosed if the post-bronchodilator (200mcg salbutamol) FEV_1/FVC ratio was <LLN at follow up. Lower limit of normal calculated using reference equations from the NHANES III study population[17,18]. Total n= those without chronic airflow obstruction at baseline who had a measurement for FEV_3/FVC ratio.*

Knox-Brown B, *et al. BMJ Open Respir Res* 2023; 10:e002056. doi: 10.1136/bmjresp-2023-002056

eTable 4. Association between baseline isolated small airways obstruction and airflow obstruction at follow-up in the UK Biobank for FEF_{25-75}.

	Total *n*	Isolated SAO (baseline) *n*	AO (follow-up) *n*	OR (95%CI)	p-value	β coefficient (95%CI)*	p-value
**Overall model**	26512	549	116	3.79 (3.10, 4.71)	<0.0001	-4.45 (-5.05, -3.85)	<0.0001
Follow-up time <5 years	5172	105	20	3.77 (2.23, 6.37)	<0.0001	-5.16 (-6.63, -3.69)	<0.0001
Follow-up time ≥ 5 years	21340	444	96	3.82 (3.01, 4.85)	<0.0001	-4.32 (-4.97, -3.66)	<0.0001
**Stratified by sex**							
Male	11197	136	44	7.75 (5.29, 11.34)	<0.0001	-5.65 (-6.88, -4.41)	<0.0001
Female	15315	413	72	2.80 (2.14, 3.66)	<0.0001	-4.04 (-4.72, -3.37)	<0.0001
**Never smoked**	15960	326	62	3.57 (2.67, 4.76)	<0.0001	-4.35 (-5.11, -3.60)	<0.0001

*Linear associations between isolated small airways obstruction for FEF_{25-75} at baseline and follow-up FEV_1/FVC ratio were estimated using mixed effects linear regression models. *Negative regression coefficient indicates a reduction in FEV_1/FVC ratio (ie, worsened lung function). Associations between isolated small airways obstruction at baseline and progression to airflow obstruction were estimated using mixed effects logistic regression models. Models were adjusted for sex, age, BMI, smoking status, follow-up duration, and smoking pack years. As there was a possibility that associations varied by testing site, we fitted a random slope model to average the associations across sites. Isolated small airways obstruction was identified if the mean forced expiratory flow rate between 25% and 75% of the forced vital capacity (FEF_{25-75}) was below the lower limit of normal (<LLN) and the pre-bronchodilator forced expiratory volume in 1 second as a ratio of the forced vital capacity (FEV_1/FVC ratio) was equal to or above the lower limit of normal (≥LLN) at baseline. Airflow obstruction was diagnosed if the FEV_1/FVC ratio was <LLN at follow up. Lower limit of normal calculated using reference equations from the NHANES III study population[17,18]. Total n= those without airflow obstruction at baseline who had a measurement for FEF_{25-75} and who had a measurement of FEV_1/FVC at follow-up.*

eTable 5. Association between baseline isolated small airways obstruction and airflow obstruction at follow-up in the UK Biobank for FEV_3/FVC ratio.

	Total *n*	Isolated SAO (baseline) *n*	AO (follow-up) *n*	OR (95%CI)	p-value	β coefficient (95%CI)*	p-value
**Overall model**	26512	162	17	1.49 (0.90, 2.47)	0.125	-0.28 (-1.41, 0.84)	0.626
Follow-up time <5 years	5172	24	4	-	-	-1.94 (-5.07, 1.18)	0.223
Follow-up time ≥ 5 years	21340	138	13	1.29 (0.73, 2.30)	0.378	-0.83 (-2.02, 0.38)	0.178
**Stratified by sex**							
Male	11197	55	7	1.70 (0.77, 3.80)	0.191	-0.81 (-2.83, 1.21)	0.431
Female	15315	107	10	1.37 (0.71, 2.65)	0.343	-1.07 (-2.41, 0.26)	0.115
**Never smoked**	15960	96	8	1.23 (0.59, 2.54)	0.576	-0.89 (-2.24, 0.48)	0.206

*Linear associations between isolated small airways obstruction for FEV_3/FVC ratio at baseline and follow-up FEV_1/FVC ratio were estimated using mixed effects linear regression models. *Negative regression coefficient indicates a reduction in FEV_1/FVC ratio (ie, worsened lung function). Associations between isolated small airways obstruction at baseline and progression to airflow obstruction were estimated using mixed effects logistic regression models. Models were adjusted for sex, age, BMI, smoking status, follow-up duration, and smoking pack years. As there was a possibility that associations varied by testing site, we fitted a random slope model to average the associations across sites. For follow-up time <5 years, the logistic regression would not converge due to the small sample size. Isolated small airways obstruction was identified if the forced expiratory volume in 3 seconds as a ratio of the forced vital capacity (FEV_3/FVC ratio) was below the lower limit of normal (<LLN) and the forced expiratory volume in 1 second as a ratio of the forced vital capacity (FEV_1/FVC ratio) was equal to or above the lower limit of normal (≥LLN) at baseline. Airflow obstruction was diagnosed if the FEV_1/FVC ratio was <LLN at follow up. Lower limit of normal calculated using reference equations from the NHANES III study population[17,18]. Total n= those without airflow obstruction at baseline who had a measurement for FEV_3/FVC ratio and who had a measurement of FEV_1/FVC at follow-up.*

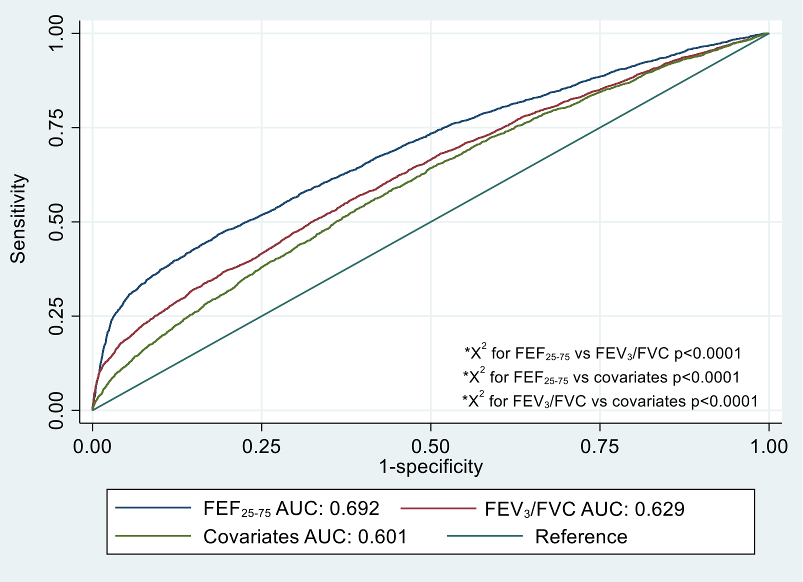

e**Figure 1.** Receiver operator characteristic curve and area under the curve (AUC) comparing ability of $FEF_{25\text{-}75}$ and FEV_3/FVC ratio to a model containing age, sex, BMI and smoking history alone to discriminate future airflow obstruction in UK Biobank participants. *P-value less than 0.05 indicates significant difference between models according to X^2 test

Knox-Brown B, *et al. BMJ Open Respir Res* 2023; 10:e002056. doi: 10.1136/bmjresp-2023-002056

eTable 6. Association between baseline isolated small airways obstruction and airflow obstruction at follow-up in the UK Biobank excluding those with asthma**.**

	**Total** *n*	**Isolated SAO (baseline)** *n*	**AO (follow-up)** *n*	**OR (95%CI)**	**p-value**	**β coefficient (95%CI)***	**p-value**
**FEF_{25-75}**	13046	212	43	3.94 (2.77, 5.60)	<0.0001	-4.17 (-5.15, -3.18)	<0.0001
**FEV_3/FVC**	13046	82	13	2.65 (1.45, 4.82)	0.001	-2.02 (-3.61, -0.43)	0.013

*Linear associations between isolated small airways obstruction for at baseline and follow-up FEV_1/FVC ratio were estimated using mixed effects linear regression models. *Negative regression coefficient indicates a reduction in FEV_1/FVC ratio (ie, worsened lung function). Associations between isolated small airways obstruction at baseline and progression to airflow obstruction were estimated using mixed effects logistic regression models. Models were adjusted for sex, age, BMI, smoking status, follow-up duration, and smoking pack years. As there was a possibility associations varied by testing site, we fitted a random slope model to average the associations across sites. Isolated small airways obstruction was identified if the mean forced expiratory flow rate between 25% and 75% of the forced vital capacity (FEF_{25-75}) was below the lower limit of normal (<LLN) or if the forced expiratory volume in 3 seconds as a ratio of the forced vital capacity (FEV_3/FVC ratio) was below the LLN and the pre-bronchodilator forced expiratory volume in 1 second as a ratio of the forced vital capacity (FEV_1/FVC ratio) was equal to or above the lower limit of normal (≥LLN) at baseline. Airflow obstruction was diagnosed if the FEV_1/FVC ratio was <LLN at follow up. Lower limit of normal calculated using reference equations from the NHANES III study population[17,18]. Total n= those without airflow obstruction or self-reported asthma at baseline, who had a measurement for FEF_{25-75} and FEV_3/FVC and who had a measurement of FEV_1/FVC at follow-up.*

Knox-Brown B, *et al. BMJ Open Respir Res* 2023; 10:e002056. doi: 10.1136/bmjresp-2023-002056

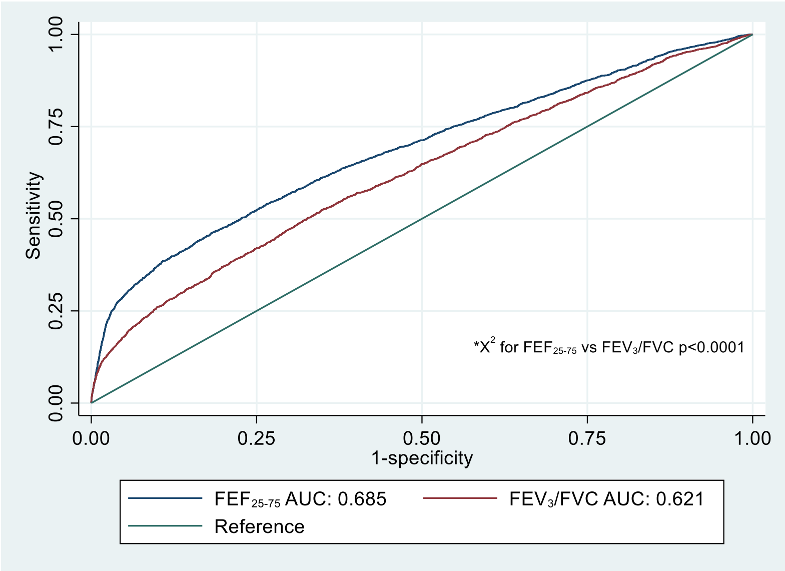

eFigure 2. Receiver operator characteristic curve and area under the curve (AUC) comparing ability of FEF_{25-75} and FEV_3/FVC ratio to discriminate future airflow obstruction in UK Biobank participants with no self-reported asthma at baseline. *P-value less than 0.05 indicates significant difference between models according to X^2 test

Brier score FEF_{25-75} = 0.1007

Brier score FEV_3/FVC = 0.1058
